# Buffalo General Hospital

**Published:** 1859-02

**Authors:** 


*Buffalo General Hospital.*—The subjoined report of the state of this institution will be most gratifying to its numerous friends and supporters, and shows a degree of prosperity which the hardness of the times did not prepare us to anticipate. We have already given an account of this institution, which will be one of the most complete hospitals in the country; from the report below, which we have copied from a daily paper, the time does not seem far distant when the whole of the original design will be completed.

*Annual Meeting of the Buffalo General Hospital Association.*—The annual meeting of this association was held on Wednesday afternoon, at the office of Charles E. Clarke, for the purpose of electing Trustees for the ensuing year, and receiving the report of the Treasurer. The following were the gentlemen elected: Charles E. Clarke, Andrew J. Rich, George S. Hazard, Henry Martin, George Howard, Bronson C. Rumsey, Jason Parker, Roswell L. Burrows, Wm. T. Wardwell.

A motion was adopted, to prepare a report of the history of the institution, to include a statement of its transactions for the year, and also the report of the Treasurer, which was presented at the present meeting and adopted. The following is the Treasurer's Report:

Buffalo General Hospital in account with Wm. T. Wardwell, Treasurer,

| | | Cr. |
|---|---|---|
| By balance from last Report, | | $6,851 93 |
| Received from Donations, | | 8,242 59 |
| " " Patients, | $191 00 | |
| " " " (Erie Co.) | 38 14 | |
| | | 229 14 |
| " " Interest on Deposits, | | 49 50 |
| | | $15,373 16 |

Dr.

Construction Account:

| | | |
|---|---|---|
| Contract, | $6,988 86 | |
| Extra work, | 2,459 84 | |
| Plumbing, &c., | 1,335 05 | |
| Boiler, | 450 00 | |
| | | $11,233 75 |

| | | |
|---|---|---|
| Furnishing account, | | 776 68 |
| Interest on mortgages, | | 544 30 |
| Insurance, | | 100 00 |
| Expenses: | | |
| Groceries and flour, | $223 07 | |
| Monthly statements of Warden, | 892 96 | |
| | | $1,116 03 |
| Medicines, liquors, &c., | | 331 88 |
| Sundry expenses, | | 8 45 |
| Printing, gas bills, &c., | | 129 30 |
| | | $14,240 39 |
| Balance to new account, | | $1,132 77 |

Wm. T. Wardwell,
Treasurer.

Buffalo, January 12, 1859.

Examined and found correct, Jan. 12, 1859.

A. J. Rich,
B. C. Rumsey,
Auditing Committee.

The present amount of liabilities is as follows:

| | | |
|---|---|---|
| Due on bond and mortgage, | | $7,740 00 |
| " sundry bills, | | 2,204 19 |
| " contract, | | 1,000 00 |
| | | $10,844 19 |
| Amount cash on hand, | | 1,132 77 |
| Subscriptions considered good, | | 1,435 00 |
| | | $2,267 77 |
| Whole amount paid from organization to date, | | 33,183 85 |
| On real estate, | $15,290 00 | |
| Less mortgage, | 7,740 00 | |
| | $7,550 00 | |
| Construction account, | 22,330 89 | |
| Furnishing account, | 854 54 | |
| Present value of property, | | $30,735 43 |
| Balance, interest, insurance, expenses, &c., | | $2,448 42 |

Wm. T. Wardwell,
Treasurer.

Buffalo, Jan. 12, 1859.

| | |
|---|---|
| Subscriptions and stock (doubtful) | $6,725 42 |

Several handsome donations have been made to the institution during the past year, including $1000 from the estate of the late Judge Waldron, and a lot of land from Hon. A. H. Tracy, on which $3000 was realized. It is to be hoped that our citizens will not lose their interest in so noble a charty, but will continue to support it liberally.

